# Resistin Regulates Pituitary Lipid Metabolism and Inflammation *In Vivo* and *In Vitro*

**DOI:** 10.1155/2013/479739

**Published:** 2013-04-18

**Authors:** F. Rodriguez-Pacheco, M. G. Novelle, M. J. Vazquez, E. Garcia-Escobar, F. Soriguer, G. Rojo-Martinez, E. García-Fuentes, M. M. Malagon, C. Dieguez

**Affiliations:** ^1^Department of Physiology, School of Medicine, University of Santiago de Compostela, 15782 Santiago de Compostela, Spain; ^2^CIBER Fisiopatología de la Obesidad y Nutrición (CIBERobn), Spain; ^3^Endocrinology and Nutrition Service, Carlos Hayà Hospital, Fundación IMABIS, 29009 Málaga, Spain; ^4^CIBER Diabetes and Metabolic Illness (CIBER-DEM), Spain; ^5^Department of Cellular Biology, Physiology and Inmunology, School of Siences, University of Córdoba, Córdoba, Spain

## Abstract

The adipokine resistin is an insulin-antagonizing factor that also plays a regulatory role in inflammation, immunity, food intake, and gonadal function and also regulates growth hormone (GH) secretion in rat adenopituitary cells cultures with the adipokine. Although adipose tissue is the primary source of resistin, it is also expressed in other tissues, including the pituitary. The aim of this study is to investigate the possible action of resistin on the lipid metabolism in the pituitary gland *in vivo* (rats in two different nutritional status, fed and fast, treated with resistin on acute and a chronic way) and *in vitro* (adenopituitary cell cultures treated with the adipokine). Here, by a combination of *in vivo* and *in vitro* experimental models, we demonstrated that central acute and chronic administration of resistin enhance mRNA levels of the lipid metabolic enzymes which participated on lipolysis and moreover inhibiting mRNA levels of the lipid metabolic enzymes involved in lipogenesis. Taken together, our results demonstrate for the first time that resistin has a regulatory role on lipid metabolism in the pituitary gland providing a novel insight in relation to the mechanism by which this adipokine can participate in the integrated control of lipid metabolism.

## 1. Introduction

Resistin, also known as found in inflammatory zone 3 (FIZZ3) is an adipocyte-derived hormone known to promote insulin resistance, impair adipocyte differentiation, and to promote inflammation [1–5] and that was originally identified in rats as a protein secreted by adipocytes that is under the control of different humoral signals and nutritional status; nutrition and metabolism regulate this adipoine. Resistin is decreased in fasting situations [6], whereas circulating resistin is increased in obese insulin resistant rodents [7] and humans [8]. Despite adipose tissue being the more relevant source of this protein, it has been recently reported that resistin is also expressed in the hypothalamus and in the pituitary gland [8, 9]. Central resistin administration appears to have a dual effect on metabolic homeostasis, first by acutely inhibiting feeding [10] and second by controlling glucose homeostasis and inducing hepatic insulin resistance [8, 11]. Recently, it has been demonstrated that central resistin regulates hypothalamic and peripheral lipid metabolism in a nutritional-dependent fashion and even that this regulation is opposite in peripheral organs in comparison with central effect [12]. The authors demonstrate that the anorectic effect of resistin is associated with the low levels of mRNA expression of orexigenic (agouti-related protein and neuropeptide Y) and the increased mRNA expression of anorexigenic (cocaine and amphetamine-regulated transcript) neuropeptides in the arcuate nucleus of the hypothalamus. Also they show that resistin exerts a nutritional status dependent inhibitory effect on hypothalamic fatty acid metabolism, as indicated by increased phosphorylation levels of both AMP-activated protein kinase and its downstream target acetyl-coenzyme A carboxylase, associated with decreased expression of fatty acid synthase in the hypothalamus. In addition, it is demonstrated that chronic central resistin infusion results in decreased body weight and major changes in peripheral expression of lipogenic enzymes, in a tissue-specific and nutrition-dependent manner [12].

In previous studies we tested the resistin effect on GH release *in vitro*. It has been demonstrated that the adipoine induces GH release in a dose fashion on adenopituitary cells culture at 4 h of exposition. This effect is even higher at 24 h of resistin treatment [13]. These results implicate the existence of resistin receptors in the pituitary, a receptor which it has not been cloned yet.

The aim of this study was to characterize the effect of acute and chronic resistin administration in the regulation of pituitary lipid metabolism. In addition since this adipokine have been reported to be involved in the inflammation process linked to obesity, we also studied its effects on the expression of the most-known proinflammatory cytokines, namely, TNF-alpha and Il-6 [14].

We have studied the expression of enzymes; fatty acid synthase, FAS; malonyl CoA decarboxylase, MCD; carnitina-palmitoil transferase, CPT-1; lipoprotein lipase, LPL; the proinflammatory cytokines interleukin 6, IL-6; tumor necrosis factor alfa, TNF-*α*.

## 2. Materials and Methods

### 2.1. Reagents

The fetal bovine serum (FBS) was obtained from Fisher Scientific Biobloce. Trizol reagent and MML-V reverse transcriptase (RT) were purchased from Invitrogen (Paisley, UK). Dulbecco's modified Eagle's medium (DMEM), collagenase from Clostridium histolyticum, deoxiribonuclease I crude lyophilized, hyaluronidase type I-S from Bovine test, Dispase II (neutral protease, grade II) from Roche Diagnostics, SL. (Barcelona, Spain). Resistin was obtained from Phoenix Pharmaceuticals Inc. (Karlsruhe, Germany); 10 *μ*g/rat dissolved in 5 *μ*L of saline.

### 2.2. Animals

Male Sprague Dawley rats (300–350 g) were housed in a temperature-controlled room, with a 12-h light, 12-h dark cycle (lights from 0800 to 2000 h). The experiments were performed in agreement with the International Law on Animal Experimentation, and the experimental protocols have been approved by the Ethics Committee of the University of Santiago de Compostela. Intracerebroventricular (ICV) cannulae chronic ICV cannulae were implanted under ketamine/xylazine anesthesia as previously described [11, 15, 16].

The correct location of the cannula in the lateral ventricle was confirmed by methylene blue staining. Animals were individually caged and allowed to recover for 1 wk before experiment. During the postoperative recovery period, the rats were handled regularly under nonstressful conditions.

### 2.3. Acute Resistin Treatment

One group of rats was fed ad libitum, and the other group was deprived of food for 12 h (nocturnal fasting). Rats then received either a single ICV injection of resistin (Phoenix Pharmaceuticals Inc., Karlsruhe, Germany; 10 *μ*g/rat dissolved in 5 *μ*L of saline) or vehicle. The rats were killed 1.5 h after injection. Treatments started at 0800 h and were carried out in the light phase.

### 2.4. Chronic Resistin Treatment

Brain infusion cannulae were stereotaxically placed into the lateral ventricle as described above. A catheter tube was connected from the brain infusion cannula to an osmotic minipump flow moderator (model 2001D or 2ML2; Alza Corp., Palo Alto, CA, USA). An sc pocket on the dorsal surface of the animal was created using blunt dissection, and the osmotic minipump was inserted. The incision was closed with sutures, and the rats were kept warm until fully recovered. The rats were then infused with either vehicle or resistin (10 *μ*g/day) for 6 d. On day 4, one group of rats was fed ad libitum, and the other group was deprived of food for the final two days. 

### 2.5. Pituitary Cell Dispersion and Culture

Isolated cells from rat anterior pituitary were obtained using an enzymatic dispersion protocol. Briefly, for each experiment, three to four anterior pituitaries were pooled, minced, and enzymatically dissociated by incubation in DMEM supplemented with collagenase 0.4%, deoxyribonuclease 0.01%, hyaluronidase 0.1%, and dispase II 0.2% at 37°C in a 5% CO_2_ atmosphere, and the tissues were mechanically dispersed every 10 minutes until a homogeneous cellular suspension was obtained. Cellular viability, as estimated by the trypan blue test, was always above 90%. 

Dispersed anterior pituitary cells were plated at a density of 300,000 cells onto 24-well culture plates in 1 mL DMEM supplemented with 10% FBS and 0.1% antibiotic-antimycotic solution.

Cells were incubated at 37°C in a 5% CO_2_ atmosphere, and medium was replaced by fresh DMEM-FBS after 48 h of culture. After a 3-d culture period, medium was removed, and cells were preincubated in 1 mL serum-free DMEM for 2 h to stabilize basal hormone secretion. Medium was then replaced with fresh DMEM containing increasing doses of resistin or the corresponding control vehicle and incubated for 4 h at 37°C. 

Medium samples were collected at the end of the experiments and were stored at –20°C until hormone determinations by RIA.

Cells in the culture plates were processed for RNA extraction as indicated below. 

### 2.6. Measurement of Growth Hormone by RIA

GH levels in culture media were measured in a volume of 25–50 *μ*L using a double antibody method and radioimmunoassay kits kindly supplied by the NIH (Dr. A. F. Parlow, NIDDK National Hormone and Peptide Program; Torrance, CA, USA). Rat GH-I-7 was labeled with ^125^I using the chloramine-T method and Iodo-Gen Pre-coated iodination tubes (Pierce, Rockford, IL, USA), respectively. Hormone concentration was expressed using the reference preparations GH-RP-2 as standards. Intra- and interassay coefficients of variation were below 6% and 9% for GH. The sensitivity of the assay was 5 pg/tube for GH. Accuracy of hormone determinations was confirmed by assessment of rat serum samples of known hormone concentrations used as external controls.

### 2.7. Real-Time Quantitative PCR of Enzymes Involved in Lipid Metabolism

The mRNA levels of acetyl-coenzyme A carboxylase (ACC)*α*, IL-6, TNF *α*, and lipoprotein lipase (LPL) were studied by using real-time PCR (TaqMan; Applied Biosystems, Foster City, CA, USA) by using specific primers and probes published as supplemental data on The Endocrine society's journals Online web site at http://endo.endojournals.org/) as previously described [17, 18]. All reactions were carried out using the following cycling parameters: 50°C for 2 min, 95°C for 10 min followed by 40 cycles of 95°C for 15 sec, and 60°C for 1 min [17, 18].

For the analysis of the data, the input value of the gene of interest was standardized to the 18S value for the sample group and was expressed compared with the average value for the vehicle-treated group. We used six to eight rats per group.

### 2.8. Statistical Analysis

Data are expressed as mean ± SEM in relation (%) to vehicle-treated rats. Statistical significance was determined by *t*-Student's test when two groups were compared or by ANOVA and post-hoc two-tailed Bonferroni test when more than two groups were compared. *P* < 0.05 was considered significant. The program used for the analysis was GraphPad Prism.

## 3. Results

### 3.1. Central Acute Administration of Resistin Does Not Regulate Pituitary Fatty Acid Metabolism

**Figure 1:**
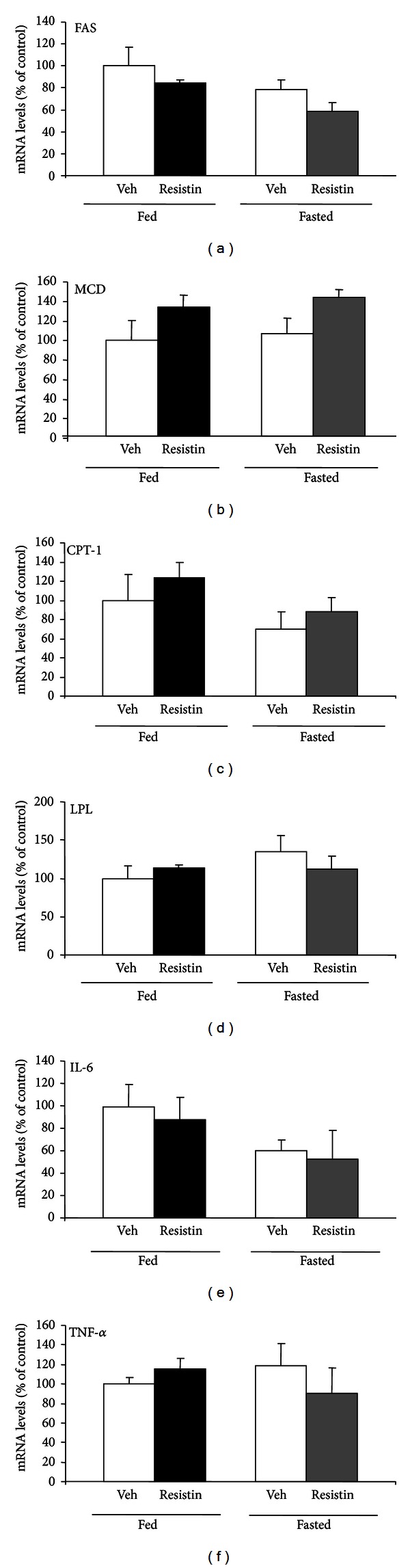
Effect of central acute administration of resistin on pituitary lipid metabolism enzymes and proinflammatory cytokines. Mean + SEM mRNA levels of FAS, MCD, CPT-1, LPL, IL-6, and TNF-alpha in the pituitary gland of fed and fasted rats following administration (I.C.V) of vehicle (Veh) or resistin (10 ug/rat). Samples were obtained 90 min later. *N* = 6–8 rats per group assay.

Central acute resistin administration induced no changes in the mRNA expression of FAS, MCD, CPT-1, LPL, and the proinflammatory cytokines IL-6 and TNF-*α* suggesting that pituitary gland fatty acid metabolism is not regulated by central acute resistin treatment (Figure 1).

### 3.2. Central Chronic Administration of Resistin Regulates Pituitary Fatty Acid Metabolism

**Figure 2:**
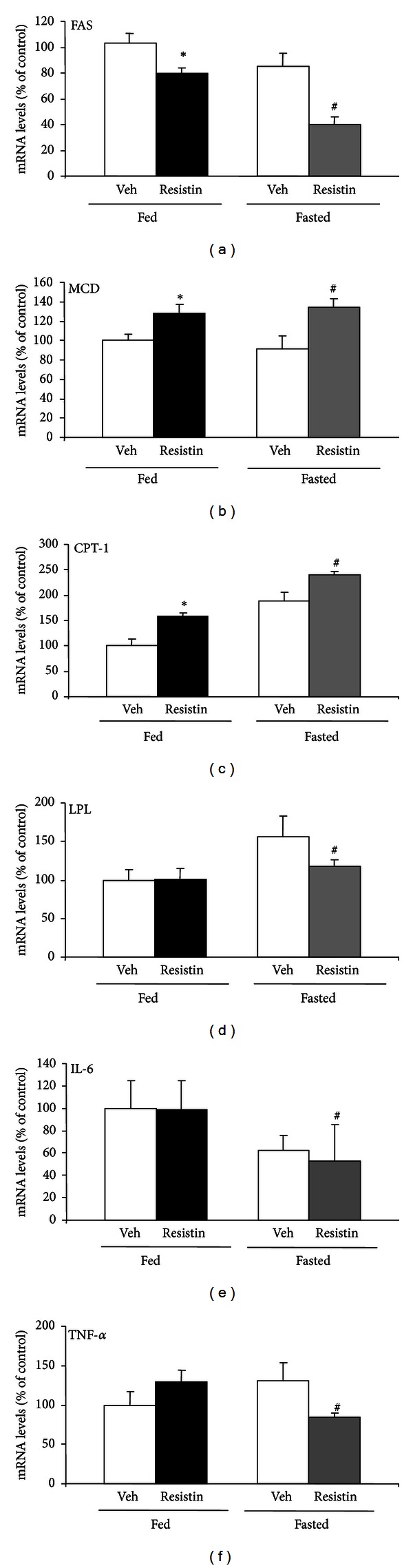
Effect of central chronic administration of resistin on pituitary lipid metabolism enzymes and proinflammatory cytokines. Mean + SEM mRNA levels of FAS, MCD, CPT-1, LPL, IL-6, and TNF-alpha in the pituitary gland of fed and fasted rats following administration (I.C.V.) of vehicle (Veh) or resistin (10 ug/day/over six days. *N* = 6–8 rats per group assay; *P < 0,05 versus fed vehicle; ^#^ *P* < 0.05 versus fasted vehicle.

Central chronic administration of resistin was associated with a marked decreased in the expression levels of FAS and LPL indicating that resistin does not participate on lipid synthesis. We also observed that CPT-1 and MCD mRNA levels are higher than in fasting conditions and in presence of resistin. With respect to the proinflammatory cytokines, resistin diminished mRNA levels of both IL-6 and TNF-*α* on fasted rats (Figure 2).

### 3.3. Effect of Resistin on Rat Pituitary Cell Cultures

**Figure 3:**
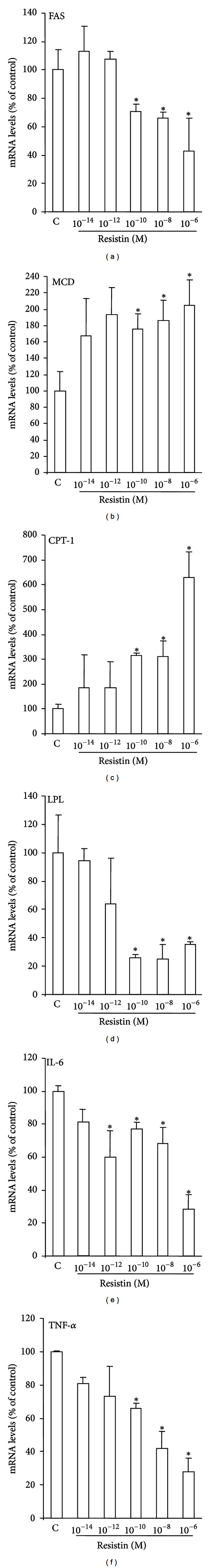
Effect of resistin on mRNA levels of lipid metabolism enzymes and proinflammatory cytokines *in vitro*. Mean + SEM mRNA levels of ACC*α*, CPT-1, FAS, LPL, MCD, SCD-1, SREBP, IL-6, and TNF-alpha. After 3 days of culture, dispersed rat pituitary cells were incubated in medium alone (C, Control) or in the presence of resistin (10^−14^–10^−6 ^M) for 4 h. After culture, cells were harvested, and receptor mRNA levels were determined by real-time RT-PCR. Enzymes band intensities were determined and adjusted by the signal intensity for HPRT. The averaged results were then calculated and expressed as a percentage of vehicle-treated control levels (100%). Data are the mean (±SEM) of three separate experiments. At least three replicate wells were evaluated per treatment in each experiment. *P < 0,05 versus corresponding control.

For knowing if resistin has a direct participation on pituitary lipid metabolism, we have examined the effect of increasing doses of resistin (10^−14^–10^−6 ^M) on mRNA levels by rat anterior pituitary cell cultures exposed to the adipokine for 4 h. As shown in Figure 3, when resistin is administered for 4 h, significantly decreased FAS shows low levels of mRNA with 10^−8 ^M and 10^−6 ^M resistin concentrations. The LPL shows decreased levels of the enzyme expression at the highest resistin doses employed. With respect to CPT-1 enzyme, resistin induces an enhanced transferase enzyme expression at 10^−8^ and 10^−6 ^M doses of the adipokine. The two proinflammatory cytokines that we have studied showed low levels of the mRNA at 10^−6 ^M. TNF-alpha even presents low levels at other resistin concentrations (10^−10 ^M to 10^−6 ^M).

## 4. Discussion

Although resistin was initially suggested to promote insulin resistance and adipocyte differentiation, recent data indicate that this hormone also plays a pleiotropic role in rodents, immunity, food intake, gonadal function, and hypothalamic and peripheral lipid metabolism regulation [19]. A recent study of our group added a new role to resistin, regulating pituitary somatotrope cell function. Resistin enhances GH release through the activation of multiple signaling pathways [13]. 

Specifically, resistin enhanced GH release on ad libitum feeding rats when resistin was administered in an acute (1.5 h) or a chronic (6 d) way increasing the transcripts numbers of the pituitary transcription factor Pit-1. It is important to confirm whether resistin-induced changes in mRNA expression of Pit-1 correlate with hormone protein levels, because it has been recently reported that POMC altered gene expression is not always linked to specific changes in *α*-MSH hypothalamic protein content [20]. Further work, using different experimental techniques as HPLC combined with RIA or proteomic analysis, will help to clarify these issues [21]. 

Previous work in our group reported that 4 h of resistin administration to dispersed rat anterior pituitary cells increased GH release [13]. Therefore, our results confirm the regulatory role of resistin on GH secretion with rats fed ad libitum. It is widely accepted that the regulation of the secretion of GH from the anterior pituitary gland is under the reciprocal control of two hypothalamic hormones, the stimulatory GHRH found in the arcuate nucleus and the inhibitory hormone, somatostatin (SRIF), synthesized in the periventricular nucleus [22]. Our results suggest that resistin might regulate GHRH or SRIF secretion to obtain GH release, or as it has been reported previously, resistin could act directly over somatotropes to regulate GH release [13]. 

That resistin regulates lipid metabolism centrally and in the periphery as has been reported by Kjems and coworkers [12]. Herein, we have expanded the research to the pituitary gland, studying if resistin regulates fatty acid metabolism in the gland *in vivo* and in adenopituitary cells cultures *in vitro*. 

We demonstrate that chronic central resistin administration during 6 d but, no acute administration during 90 min, modified the mRNA levels of the fatty acid metabolism enzymes in the pituitary gland. Besides, resistin diminished the IL-6 and TNF-*α* mRNA levels on the rat with resistin chronic treatment and on the rat adenopituitary cultures exposed to the adipokine. In detail, resistin diminished mRNA levels of FAS in the pituitary gland and enhanced mRNA levels of CPT-1 and MCD when resistin was administered during 6 d to rats, but there was no effect when the adipokine was administered in an acute way (90 min). Our data showed that central chronic resistin treatment causes a reduction in the number of transcripts of fat-promoting enzymes in the gland and that the adipokine promotes to *β*-oxidation in a nutrition-independent fashion. The physiological significance of this effect in the pituitary gland is intriguing. We confirmed that fatty acid metabolism enzymes regulation in the gland is opposite to that described by other authors in other peripheral tissues as white adipose tissue and liver [12]. The reason why resistin increases CPT-1 and MCD in pituitary and diminishes FAS expression is unclear; however, it indicates that pituitary fatty acid metabolism enzymes expression may be regulated by mechanisms distinct from those operating in adipocytes and hepatocytes. Other enzymes such as leptin also have different roles depending on whether they act at the central or peripheral level [23]. On the other hand, our results are similar in part with the results of Kjems and coworkers published on the hypothalamus in which central resistin diminished FAS mRNA levels [12].

We suggest that, as in the hypothalamus, low levels of pituitary ACC mRNA may be a compensatory physiological mechanism that prevents harmful high levels of malonyl-CoA produced in the gland after FAS inhibition [24]. Respect to CPT-1 and MCD, our results showed that CPT-1 mRNA levels are enhanced in resistin-treated fed rats within the same time frame, suggesting that malonyl-CoA levels are changed, as a consequence of ACC inactivation and MCD activation that maybe derived from the low ACC mRNA levels and the high MCD mRNA levels. 

Recent data point to the fact that central resistin induces hepatic insulin resistance by increasing the expression of proinflammatory cytokines, such as IL-6 and TNF*α*, via an unidentified mechanism mediated by the autonomic nervous system [8, 11]. Those alterations in proinflammatory adipocytokines may be due, in part, to the excess accumulation of fatty acids and triglycerides in the liver after central treatment with resistin [25–27]. Therefore, the no increase of fat deposition due to tilt toward the *β*-oxidation might at least partially explain the low mRNA levels of IL-6 and TNF-*α* on the gland after resistin treatment centrally and directly on the adenopituitary cells cultures. Further investigation is necessary to clarify this issue.

Finally, we investigated the effect of resistin on lipid metabolism on pituitary cells cultures, and we found the same result observed *in vivo, *resistin diminished the mRNA levels of the enzymes involved in regulating triglyceride uptake and lipid metabolism, such as LPL, ACC, FAS, SCD-1, and key transcriptional factors regulating lipid metabolism, such as SREBP-1c and enhanced the transcripts numbers of CPT-1 and MCD when resistin was administered during 4 h on adenopituitary cells cultures. We found significant activity at resistin 10^−12^ and resistin 10^−10 ^M, which is within the concentration range of circulating resistin in rat [28]. Noteworthy, the effect of resistin did not follow a typical dose-response pattern in receptor gene expression, an observation that has been reported for this and other signalling molecules [29–32]. Although the reasons are unclear at present, several possibilities can be put forward. It is possible that, depending on its concentration, resistin might induce different structural conformations of its receptor(s) and/or the selective interaction of resistin receptor(s) with other receptors (i.e., homo- or heterodimerization), which in turn might modify resistin activity as it has been reported to occur for other receptors in response to their corresponding ligands [33]. Anyhow, it is yet unknown whether the effects of resistin are mediated by one or several receptors because none has been identified. If there is more than one receptor for resistin, it could be possible that they may have different affinities for resistin with different biological effects that could explain the atypical dose-response curves observed herein. However, until more data, these explanations are presently speculative. Resistin could be inducing to *β*-oxidation against to promote fat storage. Other peptides as ghrelin or GHRH being GH secretagogues have a similar behaviour on lipid metabolism in the pituitary (preliminary data from our laboratory). Besides, resistin diminished the IL-6 and TNF-*α* mRNA levels on rat adenopituitary cultures exposed to the adipokine as occured in the *in vivo* experiments. These results show that resistin promotes the *β*-oxidation in the pituitary gland, decreasing the levels of proinflammatory cytokines and that the adipokine does this action directly on anterior pituitary cells, showing that the pituitary gland could be a target in the control of diseases caused by insulin resistance.

In summary we show that administration of resistin to rats *in vivo* and to adenopituitary cell cultures *in vitro* evoked important effects on pituitary.

Chronic infusion of central administration of resistin increased enzymes mRNA levels implicated on lipid *β*-oxidation activity and inhibited mRNA expression levels of the enzymes involved on lipid synthesis* in vivo* and *in vitro.*

Chronic administration of the adipokine decreased IL-6 and TNF-*α* transcripts numbers *in vivo* and on primary cultures experiments.

When viewed together, these results provide evidence that pituitary is a direct and indirect target of resistin action for lipid metabolism regulation of the gland itself. It is feasible that pituitary adipokine expression serves as a link between peripheral metabolic signals. Pituitary control fat storage and metabolism could provide another important step in unraveling the interactions between center, periphery, and adipocytokines, which will improve our understanding of metabolic syndrome and obesity.
